# Supplementary material for: Suppression of Smoldering of Calcium Alginate Flame-Retardant Paper by Flame-Retardant Polyamide-66
Source: Polymers (Basel). 2021 Jan 29;13(3):430. doi: 10.3390/polym13030430 (PMC7866267; doi:10.3390/polym13030430)
Supplement: Supplementary file 1 [file polymers-13-00430-s001.pdf]

polymers

MDPI

Article

Suppression of Smoldering of Calcium Alginate Flame-Retardant Paper by Flame-Retardant Polyamide-66

Kai Xu [1,2], Xing Tian [1], Ying Cao [2], Yaqi He [2], Yanzhi Xia [1] and Fengyu Quan [1,2,*]

1 State Key Laboratory of Bio-Fibers and Eco-Textiles, Collaborative Innovation Center of Marine Biobased Fiber and Ecological textile Technology, Institute of Marine Biobased Materials, Qingdao University, Qingdao 266071, China; 2018020388@qdu.edu.cn (K.X); xingtian1982@qdu.edu.cn (X.T.); xyz@qdu.edu.cn (Y.X.)
2 College of Materials Science and Engineering, Qingdao University, Qingdao 266071, China; 2019020444@qdu.edu.cn (Y.C.); 2019204670@qdu.edu.cn (Y.H.)
* Correspondence: quanfengyu@qdu.edu.cn

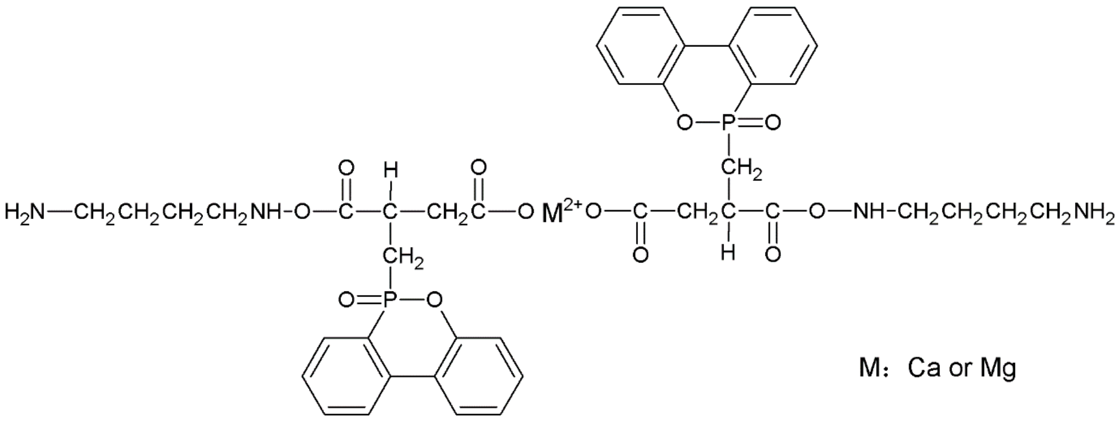

Figure S1. Structural formula of phosphorus flame retardant in FR-PA fiber.

Table S1. Composition of the prepared paper samples.

	Wood Pulp (wt%)	FR-PA Pulp (wt%)	Ca-Alg Pulp (wt%)
Wood pulp paper	100	0	0
Ca-Alg paper	0	0	100
FR-PA/Ca-Alg (10/90)	0	10	90
FR-PA/Ca-Alg (20/80)	0	20	80
FR-PA/Ca-Alg (30/70)	0	30	70
FR-PA/Ca-Alg (40/60)	0	40	60

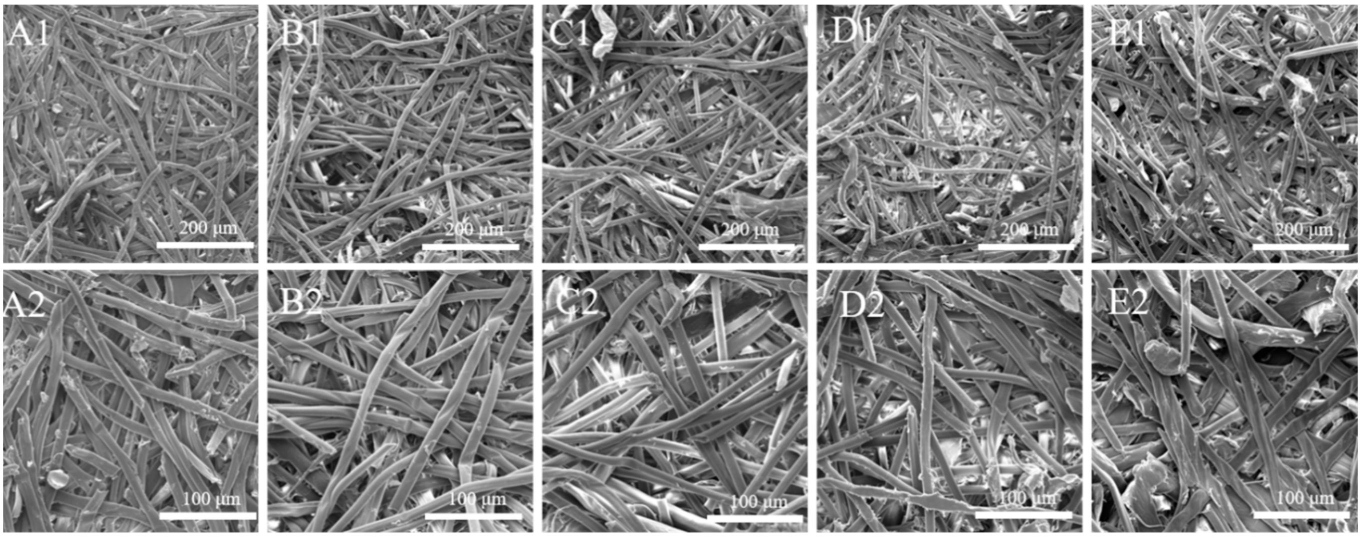

Figure S2. SEM photographs of Ca-Alg paper (A1, A2), FR-PA/Ca-Alg (10/90) paper (B1, B2), FR-PA/Ca-Alg (20/80) paper (C1, C2), FR-PA/Ca-Alg (30/70) paper (D1, D2) and FR-PA/Ca-Alg (40/60) paper (E1, E2) at different magnification (×500, ×1000).

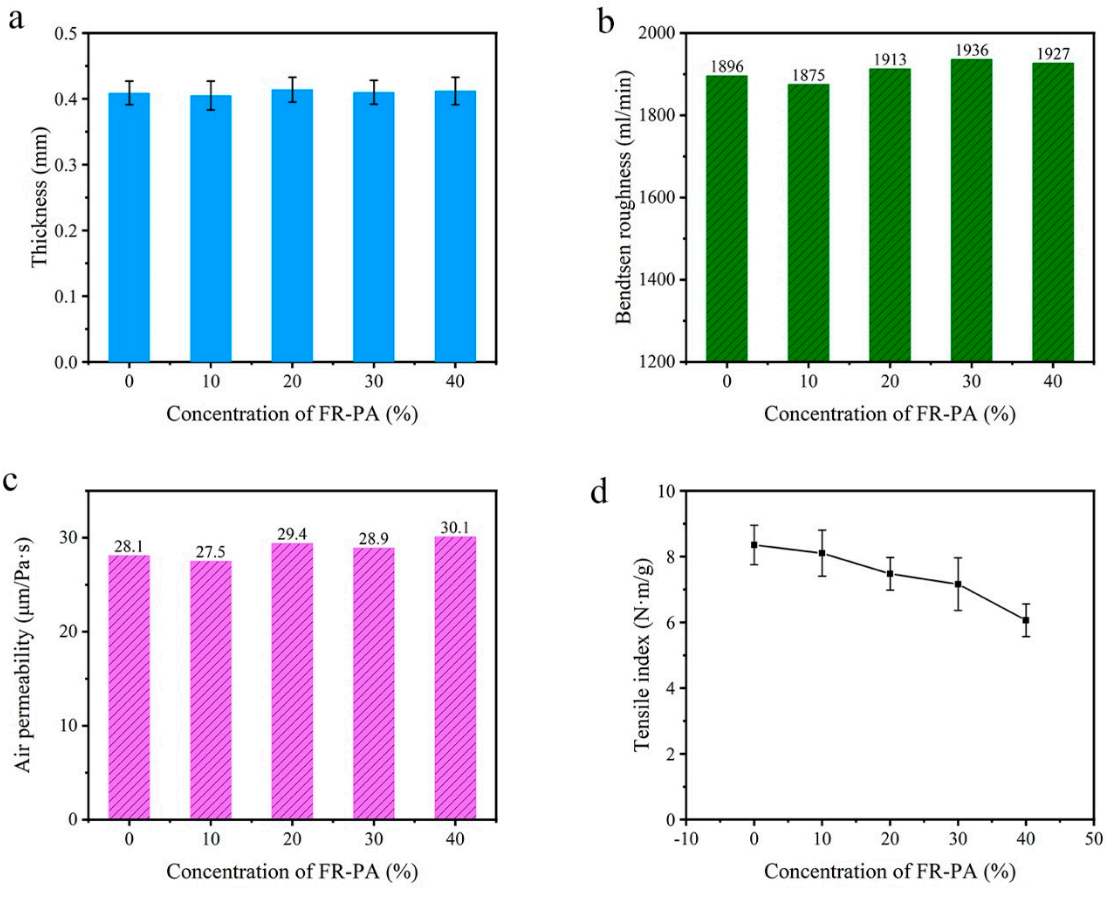

Figure S3. Physical properties of prepared paper: (**a**) thickness, (**b**) Bendtsen roughness, (**c**) air permeability and (**d**) tensile index of Ca-Alg and composite papers.

As shown in Figure S3a, the thickness of Ca-Alg, FR-PA/Ca-Alg (10/90), FR-PA/Ca-Alg (20/80), FR-PA/Ca-Alg (30/70) and FR-PA/Ca-Alg (40/60) paper is 0.409, 0.405, 0.414, 0.410 and 0.412 mm, respectively, which is almost the same. Figure S3d shows the tensile index Ca-Alg and composite papers. it can be seen that the tensile index of composite papers decrease gradually as the FR-PA concentration increases. The tensile index of the Ca-Alg paper is 8.35 Nm/g,

whereas the tensile index of FR-PA/Ca-Alg (30/70) composite paper is 7.16 Nm/g, 14.25% lower, indicating that FR-PA reduces the tensile strength of the composite paper, but the change value is not large in the case of no adhesive. The tensile strength of composite paper can be improved by adding a binder.
